# Study of the Electrical Properties of Aluminate Cement Adhesives for Porcelain Insulators

**DOI:** 10.3390/ma14092232

**Published:** 2021-04-26

**Authors:** Huiwen Wan, Zhangyin Hu, Gang Liu, Jiadong Xiao, Yong Wang

**Affiliations:** 1 School of Materials Science and Engineering, Wuhan University of Technology, Wuhan 430070, China; wanhw@whut.edu.cn; 2 State Key Laboratory of Silicate Materials for Architectures, Wuhan University of Technology, Wuhan 430070, China; hzy1022@whut.edu.cn; 3 School of Civil Engineering and Architecture, Wuhan University of Technology, Wuhan 430070, China; xiao_jiadong@whut.edu.cn; 4 College of Water and Architectural Engineering, Shihezi University, Xinjiang 832003, China; wyong@shzu.edu.cn

**Keywords:** aluminate cement, silica fume, resistivity, saturated state, unsaturated state

## Abstract

Electrical properties are one of the essential parameters of cement-based materials used in suspension porcelain insulators. This paper studied the electrical properties of aluminate cement adhesives (ACA) containing silica fume (SF), as well as their compressive strength and porosity. The results indicated that the addition of silica fume improved the resistivity of ACA under a saturated state (relative humidity is 50%). This was mainly attributed to the decrease of the ACA’s pore connectivity due to the SF’s filling effect. However, the early compressive strength of ACA was slightly reduced by the addition of SF. Under an unsaturated state, the ACA’s resistivity without the SF gradually exceeded that with the SF at the extension of drying time. The nuclear magnetic resonance (NMR) results indicated that the addition of SF content increased the ACA’s porosity; for the tiny pores especially, (the size less than 25 nm), this increased by 3.4%. Meanwhile, the addition of SF increased the tortuosity of the ACA’s conductive channels, which could improve its resistivity. Therefore, SF is recommended to be used in cement-based adhesives on insulators to lower the cost and improve the resistivity.

## 1. Introduction

Electrical properties are one of the essential parameters of cement-based materials [1]. In recent years, there has been more and more research on the resistivity of cement-based materials. On the one hand, the cement needs to have high resistance properties in some practical applications, such as for cement adhesive in suspension porcelain insulators [2]. Due to the unique working conditions (high-voltage lines), the higher the resistance, the better. On the other hand, some studies have shown that the electrical conductivity of cement-based materials is closely related to the permeability, durability, freezing resistance and other properties of the material, generally considered to be an essential parameter for research and evaluation of these properties [3,4,5]. The reason is that the conductivity of cement-based materials depends on the pore characteristics (porosity, pore connectivity, etc.), and the pore characteristics are important factors that affect its durability, permeability and other properties [6]. Additionally, electrical properties are often used to study the early behavior of cement [7,8,9], such as the early hydration behavior of cement slurry by measuring the material’s resistivity within 48 h [9].

Compared with Portland cement, aluminate cement has many advantages such as high early strength, high-temperature resistance, corrosion resistance and strong sulfate resistance [10]. It is widely used in various projects with high early strength requirements, and heat-resistant concrete production [11]. For example, in the process of automatic and intelligent production of suspended porcelain insulators, the adhesive must have the characteristics of a fast setting time and high early strength. However, aluminate cement also has an obvious shortcoming: the hydration product structure is unstable, and the crystal form transformation is prone to occur at high temperatures or with time, resulting in post-strength shrinkage [10]. Many scholars have researched how to solve this problem, such as mixing mineral admixtures (such as slag, fly ash, silica fume, limestone powder, etc.) into the aluminate cement system to improve the problem of its post-strength reduction [12,13,14,15]. The results showed that mineral admixtures could be suppressed to a certain extent on the crystal transition aluminate cement, certainly playing a helpful role in the late stability strength.

However, in the above studies on the modification of aluminate cement by mineral admixtures, most of the research only involves the mechanical properties of aluminate cement, and there is little research on the influence of silica fume (SF) on the electrical properties of aluminate cement. On the other hand, the research on cement adhesives is limited to its mechanical properties, and seldom involves its insulation properties at present, which clearly increases electrical safety hazards when the cement adhesives work.

In this paper, aluminate cement is used as a binder for porcelain insulators, and its resistivity variation under saturated and unsaturated conditions was studied. Simultaneously, an appropriate amount of SF (8 wt.%) was added to the aluminate cement system, and the effect of SF on the compressive strength of aluminate cement adhesives (ACA) and the mechanism of action on the conductivity of ACA were discussed. Additionally, a conductive model suitable for ACA used in porcelain insulators was proposed based on the results and the structural model of porcelain insulators. The results were expected to improve the theoretical basis used to prepare cement adhesives with high insulation.

## 2. Materials and Methods

### 2.1. Materials

Two different mixtures were studied in this paper, namely Group C (pure aluminate cement) and Group S (incorporating 8% mass fraction of silica fume instead of aluminate cement). The chemical composition of aluminate cement and silica fume are shown in Table 1, respectively. The fine aggregate uses 80–140 mesh fine sand, and the water-reducing agent uses polycarboxylate superplasticizer. The experimental specimens were produced with a constant weight ratio of aluminate cement: water: fine aggregate: water-reducing agent as 1:0.18:0.33:0.0015.

### 2.2. Testing Methods

#### 2.2.1. Compressive Strength

Paste cubes measuring 40 mm × 40 mm × 40 mm were used for compressive strength tests according to ASTM C39 [16]; three parallel test blocks were used for each set of specimens. The cement, fine aggregate and silica fume need to be mixed and dry stirred for 3 min to better disperse the silica fume. The specimens were molded and cured at an ambient temperature of 20 ± 1 °C and a relative humidity of ≥95%. After curing to scheduled age, the specimen was placed in an environment with an ambient temperature of 20 ± 1 °C for compressive strength testing, and the loading rate of testing was 2.4 kN/s.

#### 2.2.2. Measurement of the Electrical Resistivity of Cement Pastes

**Figure 1:**
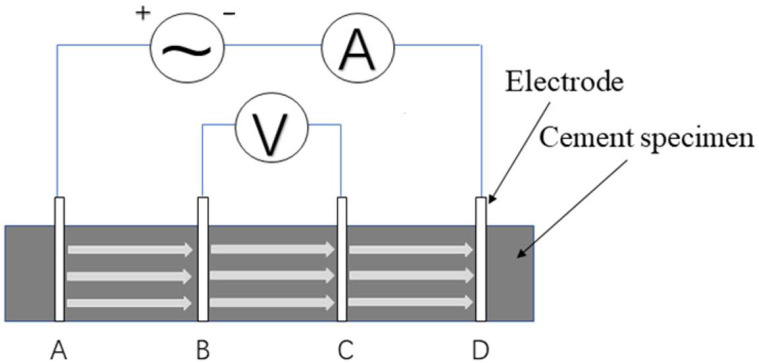
Four electrode method schematic diagram.

**Figure 2:**
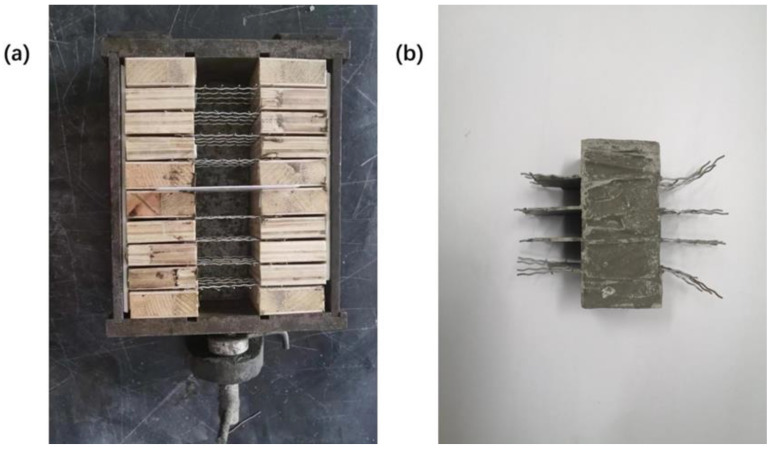
(**a**) specimen mold (**b**) embedded four electrode cement specimens.

Cement concrete conducts electricity mainly through the movement of free ions in the pore solution. At the circuit electrode, ions in the pore solution will migrate under the action of the electric field, and the electrochemical reaction with the metal electrode will result in the change of ion concentration in the pore solution, thus causing the instability of the measurement results [17]. This error can be effectively avoided by using the four-electrode method [18]. This circuit diagram is shown in Figure 1. The resistance between electrodes B and C is measured to minimize the error caused by the polarization reaction of electrodes A and D. In addition, the method of pre-embedded electrodes is used to reduce the error caused by contact resistance. At the same time, in order to avoid the deviation or tilt of the electrode mesh in the cement mortar molding process, the triple 40 mm × 40 mm × 160 mm mortar test piece mold is improved, as shown in Figure 2a.

#### 2.2.3. Moisture Content

The moisture content (MC) of ACA was calculated by the following formula:

(1) $$\frac{MC}{MC_{@100RH}}=\frac{m_{t}-m_{0}}{m_{s}-m_{0}}$$

where $\frac{MC}{MC_{@100RH}}$ is the moisture content normalized by the moisture content at 100% relative humidity (RH), $m_{t}$ is mass of specimens exposed to t days in dry environment,$\;m_{s}$ is mass of specimens at saturation state, and $m_{0}$ is mass of specimens when absolutely dry (dry to constant weight at 105 °C).

#### 2.2.4. Low-Field ^1^H NMR

In this paper, bulk relaxometry (transversal relaxation time $T_{2})$ was used to measure the porosity distribution of the cement specimens (the Meso MR12-060H-1 manufactured by Niumag Corporation (Suzhou, China) was used for nuclear magnetic resonance testing). The samples were selected from the 28 d compressive strength test block. Before the test, the samples were subjected to a vacuum pressurized saturated water treatment for 12 h. Low field nuclear magnetic resonance (NMR) resonant frequency is 23.04 MHz, the magnet temperature control was 32 ± 0.01 °C, and the probe diameter was 25 mm.

## 3. Results and Discussion

### 3.1. Surface Dry Saturated Conditions

#### 3.1.1. Compressive Strength

**Figure 3:**
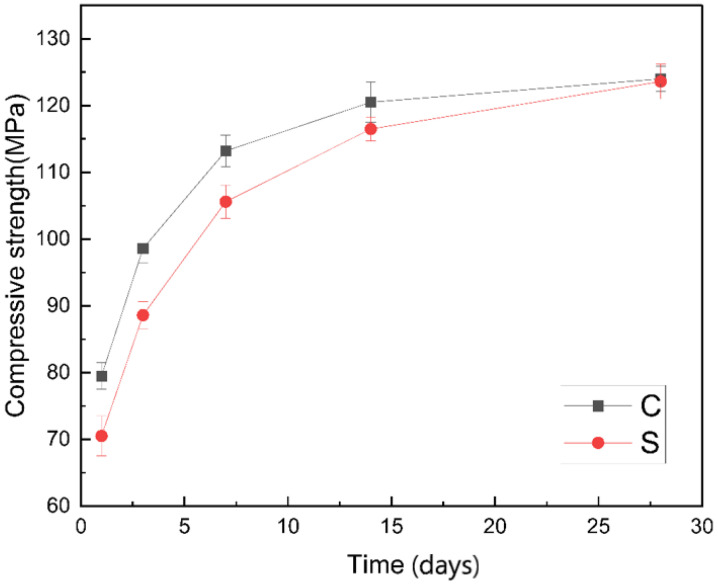
Comparison of compressive strength between control and SF8 within 28 days.

Figure 3 shows the variation of the compressive strength of the two groups over time. It can be seen that the compressive strength of group C was always higher than that of group S within the 28 days. With the extension of time, the strength difference between the two groups began to get smaller and smaller, and the compressive strength of the two groups was equal at 28 days. Using silica fume to replace cement partially will reduce hydration products. Moreover, the aluminate hydration product did not have calcium hydroxide, and the basicity of the pore solution was low, so the potential activity of the silica fume was not stimulated, and it did not participate in chemical reaction at the early stage, but only played the role of physical filling. As a result, the early strength of group S was lower than that of group C. In addition, some studies have shown that silica fume can effectively inhibit the crystalline transformation of metastable hydration products [12], which is the reason why the compressive strength of group S caught up with group C in the later stage.

#### 3.1.2. Electrical Resistivity

**Figure 4:**
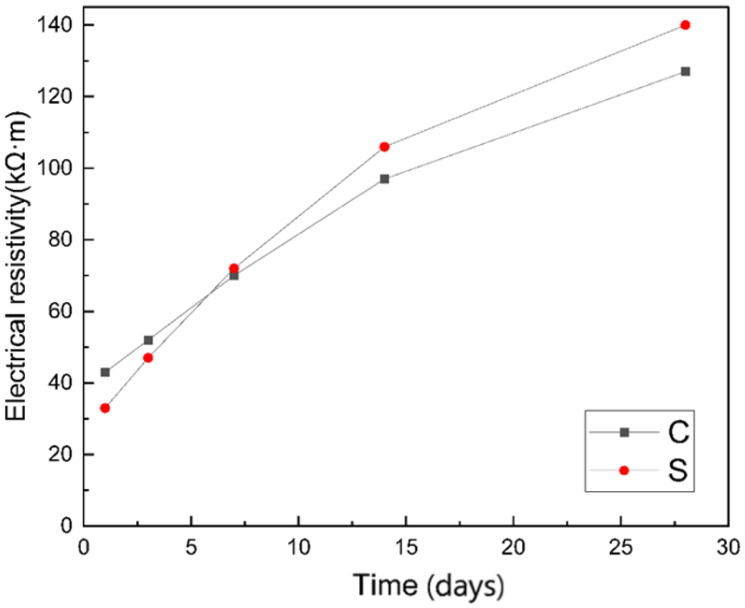
Comparison of electrical resistivity between group C and group S within 28 days.

The resistivity changes of the two groups of specimens in the saturated-surface-dried within 28 days are shown in Figure 4. The resistivity increases with the prolongation of curing time. The resistivity of group S was lower than that of group C before curing for 7 days, and then exceeded that of group C. The following will explain this change in detail through the development of early cement pores and pore channels.

**Figure 5:**
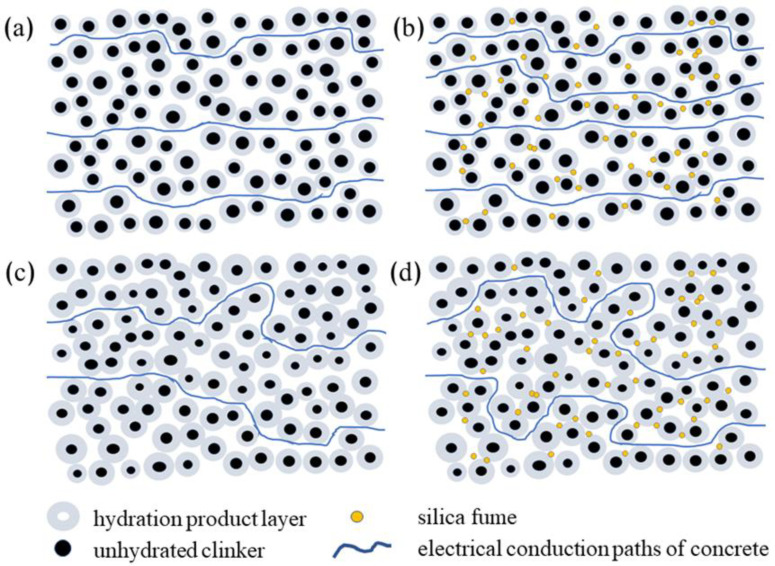
Schematic diagram of electrical conduction paths of concrete; (**a**) group C at 1 day; (**b**) group S at 1 day; (**c**) group C at 7 day; (**d**) group S at 7 day.

The schematic diagram of the pore structure in the early stage of cement hydration is shown in Figure 5. As shown in Figure 5a, the hydration degree of cement is lower at one day (compared with 28 days). There are a large number of non-hydrated particles in the structure, resulting in a large porosity and high pore connectivity. According to the previous theoretical knowledge, cement-based materials’ electrical conductivity under saturation state is positively correlated with porosity and pore connectivity. Also, the resistivity of group S was only 71% of that of group C at one day. The reason is that silica fume does not participate in the hydration of aluminate cement at the early stage, but only plays a filling role, which is not apparent under the premise of high pore connectivity (as shown in Figure 5b). Moreover, the use of silica fume to replace a part of cement will reduce the overall hydration products, resulting in larger pores formed by the hydration product overlaps.

The microstructure simulation diagram of cement hydration for seven days is shown in Figure 5c. After seven days of curing, the continuous hydration leads to a closer bond between hydration products, resulting in lower overall porosity of the material and lower pore connectivity. The tortuosity of the conductive channel is significantly increased. Group C’s resistivity was increased by 52%, and group S increased by 118%, compared with that on day 1. Also, the filling of silica fume becomes critical for the resistivity of the material since the structure is relatively dense. As shown in Figure 5d, silica fume particles with small particle sizes fill in the gap of hydration products, resulting in an increase in the tortuosity of the material’s conductive channels and a decrease in conductive channels. Although the compressive strength of group S was lower than that of group C, the resistivity exceeded that of group C.

More sufficient hydration is obtained after 28 days continuous curing. It is noted that the main hydration products CAH_10_ and C_2_AH_8_ of aluminate cement at about 20 °C are in a metastable state, and they will inevitably transform into a stable state with time or at high temperature [19]. The decrease of solid volume accompanies the crystal transformation, and the transformation process is shown as follows [20,21]:

3CAH_10_ → C_3_AH_6_ + 2AH_3_ + 18H (2)

3C_2_AH_8_ → 2C_3_AH_6_ + AH_3_ + 9H (3)

The decrease of solid phase volume accompanies the crystal transformation. At this time, group S’s resistivity reaches 110% of that of group C. However, the compressive strength of the two groups remains the same, indicating that silica fume plays a positive role in improving the late resistivity of aluminate cement.

**Figure 6:**
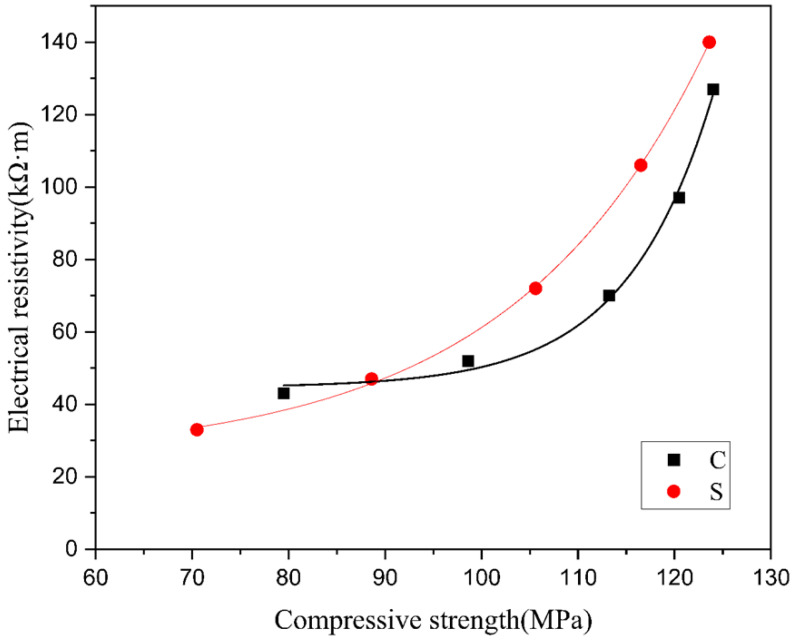
Electrical resistivity as a function of the compressive strength.

To study the connection between the compressive strength and resistivity of aluminate cement under a saturated state, data fitting was conducted for the experimental results of the two groups. As shown in Figure 6, the resistivity of both groups increased at the increase of their compressive strength, and this became more apparent when the strength was higher than 100 MPa. Based on the trend line in Figure 6, group S had a higher strength than group C under the same compressive strength.

### 3.2. Unsaturated State

#### 3.2.1. Moisture Content

**Figure 7:**
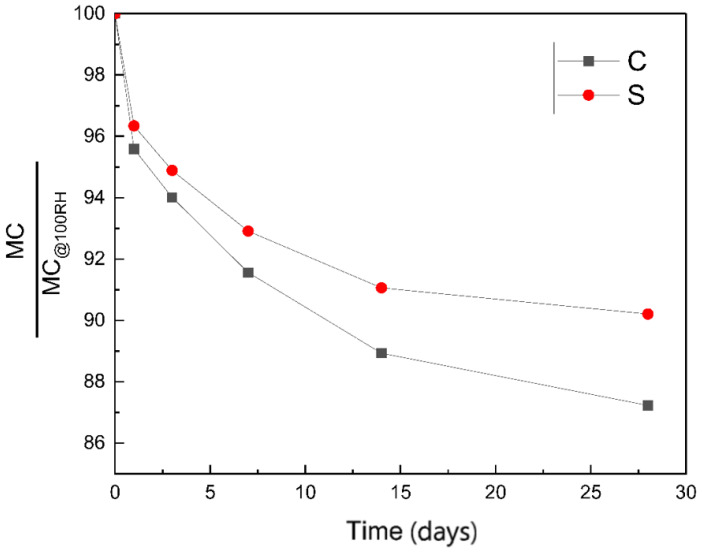
Comparison of moisture content between group C and group S within 28 days in a dry environment.

After curing for 28 days, the two groups of specimens were placed in a curing box with a relative humidity of 50% and a temperature of 20 ± 1 °C for further curing for 28 days, during which the moisture content and resistivity of the specimens were recorded. Figure 7 shows the change of material moisture content within 28 days. The moisture content of both specimens decreased significantly in a short period and then tended to be stable.

**Figure 8:**
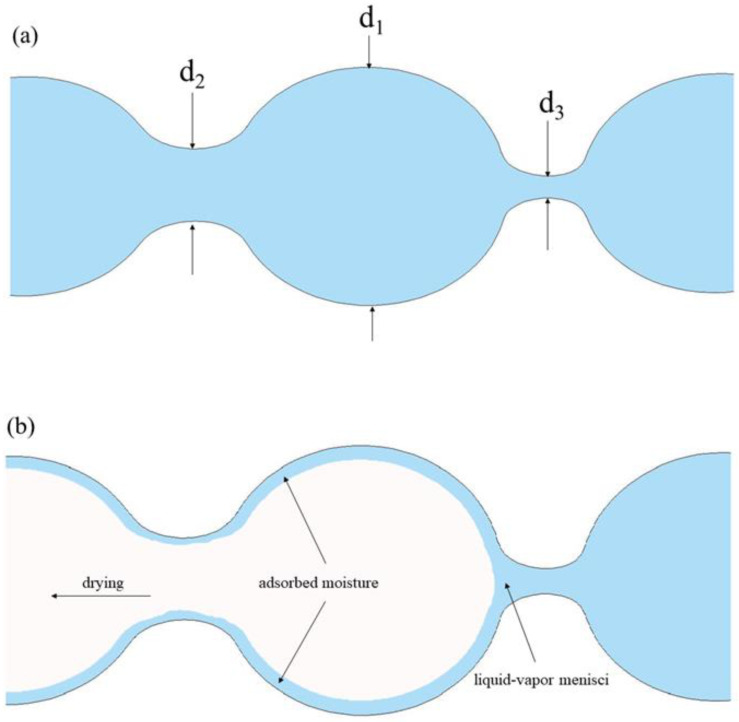
Schematic diagram of pore solution changing with environmental humidity; (**a**): saturated and (**b**): desorption.

Figure 8 shows the process of water loss in the pores of a porous material in a dry environment (relative humidity is 50%). The microscopic structure shown in the figure includes three pore sizes: a large pore with diameter d_1_, a small pore with diameter d_3_ (critical saturation), and a medium pore with diameter d_2_, which is between d_1_ and d_3_. When the saturated system is exposed to an environment with a relative humidity of less than 100%, moisture begins to evaporate from the exposed surface, with moisture in the outermost micropores first to evaporate (except for the adsorbed moisture layer). With the extension of exposure time, moisture in the medium pore (d_2_) connected with it and the large pore (d_1_) behind it also evaporates successively, and the critical saturated pore (d_3_) continues to maintain the saturation state. The large pore behind the pinhole will also remain saturated, as its water can only be drained through the pinhole [22].

We divided the material into layers from the outside to the inside. Starting from the outermost layer, the relative ambient humidity of each layer will be higher than that of the previous layer due to the ink-bottle effect of the small pores. According to the Kelvin equation [23], the critical saturated pore size is proportional to the relative ambient humidity. The critical saturated pore size of the material gradually increases from the outside to the inside, and moisture is more difficult to evaporate from the inside, which is manifested as the relative humidity in the material is significantly higher than the ambient humidity, and there is a humidity gradient.

As can be seen from Figure 7, the moisture content of group S changes more slowly than that of group C, and the moisture content of group S after 28 days of exposure is also significantly higher than that of group C. Combined with the above analysis, it can be seen that under the same environmental humidity, the change of moisture content in the material depends on the number and size of holes. This also proves from the side that adding a proper amount of silica fume in the aluminate cement system causes the material to have more holes and smaller pore sizes.

#### 3.2.2. Conductive Model of Cement Adhesive for Porcelain Insulators

The study of the conductivity of porous media in a saturated state can be traced back to 1941. Archie studied the relationship between saturated sandstone porosity and permeability and formation resistivity factors, and established a simple relationship between formation resistivity and salt water resistivity [24].

(4) $$R_{0}=FR_{W}$$

where $R_{0}$ is the resistivity of sandstone wholly saturated with salt water, $R_{W}$ is the resistivity of salt water, and F is the resistivity factor of formation.

Generally, Hardened cement paste is roughly composed of three phases: a solid framework composed of hydrated products and non-hydrated particles, a liquid phase in the pores, and pore gas [22]. For the cement-based material in the saturated state, the pores are almost filled by the liquid phase. The resistivity of the liquid phase is several orders of magnitude lower than that of the gas and solid phases [22]. Therefore, the electrical conductivity of the cement-based material in the saturated state can be expressed by

(5) $$\sigma =\Phi^{m}\sigma_{p}$$

where σ is the conductivity of cement-based materials, $\sigma_{p}$ is the pore solution’s conductivity, Φ is the total porosity, and m is the Archie exponent.

In practical application, most cement-based materials are exposed to the air or the environment where humidity is below 100%. The existence mode of pore solution changes in this unsaturated state. Pores with different pore sizes will lose water one after another, and those with smaller pore sizes can maintain the saturation state. The electrical conductivity of cement-based materials in the unsaturated state can be expressed as follows:

(6) $$\sigma =\Phi^{m}\sigma_{p}S^{n}$$

where S is the saturation of cement-based materials, and n is the secondary Archie exponent [6,25].

**Figure 9:**
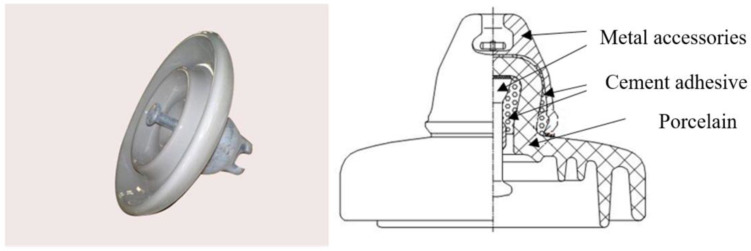
Diagram of porcelain insulator.

Equation (6) shows the conductive model of cement-based materials under unsaturated conditions. Because of the saturation gradient inside the material, the conductivity calculated by Equation (6) must be modified. Some studies have expressed multiphase materials’ conductivity as the weighted sum of the conductivity of multiphase materials [26]. Similarly, different saturation ranges of materials can be regarded as different conductive phases, so the conductivity model of materials can be expressed as follows:

(7) $$\sigma =\overset{N}{\underset{i=1}{\sum }}\Phi_{i}^{m}\sigma_{i}{S_{i}}^{n}$$

where $\sigma_{i}$,$\;\Phi_{i}$ and $S_{i}$ are the pore solution, porosity and saturation of each constitutive component, and N is the number of conductive phases. Figure 9 shows the physical drawing and section drawing of the porcelain insulator. Due to the unique structure of the porcelain insulator, only one side of the cement adhesive is in contact with the outside air. Assuming that the saturation in the material has a simple linear relationship with the length L:

(8) $$S=S_{W}+\left(\frac{S_{T}-S_{W}}{l_{0}}\right)l$$

where $S_{W}$ is the saturation of the outermost layer, $S_{T}$ is the saturation of the innermost layer, and $l_{0}$ is the length of the cement-based materials.

With the evaporation of moisture, the ionic resistivity of the pore solution decreases with the increase of concentration. However, the equivalent conductivity (K) decreases slightly with increasing ion concentration due to ion-to-ion interactions [1,22]. Due to the interaction of these two factors, the resistivity of the pore solution changes little. To facilitate the analysis, this paper takes the ion concentration of pore solution as the quantitative quantity. Meanwhile, it is assumed that the material is uniform and the porosity of each layer is consistent. Combined with Equations (6) and (8), the electrical conductivity of each layer of the material can be expressed as

(9) $$\sigma =\Phi^{m}\sigma_{p}{\left[S_{W}+\left(\frac{S_{T}-S_{W}}{l_{0}}\right)l\right]}^{n}$$

The resistivity is the reciprocal of the conductivity. The resistance of each layer of the material can be expressed as

(10) $$dR=\frac{dl}{\sigma A_{s}}$$

where $A_{s}$ is the cross-sectional area of the materials. The material can be regarded as numerous layers of thin slices with different saturation in series. Combined with Equations (7), (9) and (10), the resistance of the material can be expressed as

(11) $$R=\frac{1}{\Phi^{m}\sigma_{p}A_{s}}\int_{0}^{l_{0}}{\left[S_{W}+\left(\frac{S_{T}-S_{W}}{l_{0}}\right)l\right]}^{-n}dl$$

where $l_{0}$ represents the thickness of the specimen. Through a simple integral operation, the electrical resistance and conductivity of the cement adhesives used for porcelain insulators can be calculated as

(12) $$R=\frac{l_{0}\left(S_{\omega }^{1-n}-S_{T}^{1-n}\right)}{\Phi^{m}\sigma_{p}A_{s}\left(n-1\right)\left(S_{T}-S_{W}\right)}$$

(13) $$\sigma =\frac{\Phi^{m}\sigma_{p}\left(n-1\right)\left(S_{T}-S_{W}\right)}{\left(S_{W}^{1-n}-S_{T}^{1-n}\right)}$$

#### 3.2.3. The Resistivity of Unsaturated Cement-Based Materials

During the drying process, the conductive structure of cement-based material changes. Due to moisture evaporation, the interior of large and medium-sized pores can be regarded as insulating phases. However, there is an adsorbed moisture layer on the surface of pores, which can still be considered as a conductive phase to some extent. Secondly, the concentration of ions in the remaining solution is bound to increase, as moisture is removed during the drying process. The resistivity of the pore solution decreases significantly with the evaporation of moisture. In general, the liquid volume and connectivity of cement-based materials under unsaturated conditions decreases, but the ionic concentration of the solution increases, which is generally shown as an increase in resistivity.

**Figure 10:**
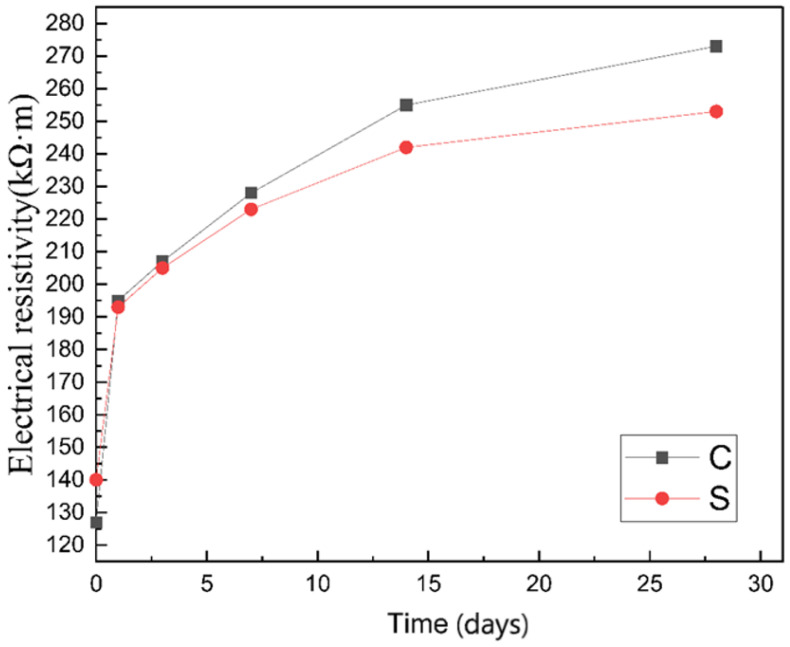
Comparison of electrical resistivity between group C and group S within 28 days in a dry environment.

The specimen’s resistivity variation trend was placed in a dry environment (50% relative humidity) for 28 days as shown in Figure 10. The changing trend of resistivity is similar to that of moisture content, which first increases significantly in a short period, then grows slowly until it flattens out. Meanwhile, group C’s resistivity exceeded that of group S after drying for 1 day, and the difference between the two groups became more and more significant with time.

**Figure 11:**
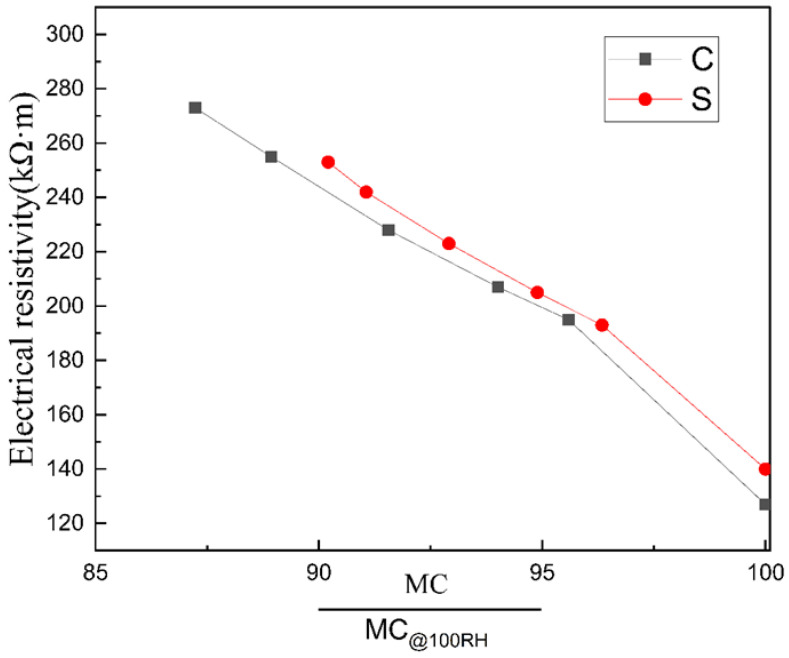
Electrical resistivity as a function of the moisture content.

Figure 11 shows the isotherm of conductivity as a function of moisture content. In general, the conductivity and water content of the material shows an excellent linear relationship at high water content (except at the beginning when the water content drops from 100%, which may be caused by a change in the conductive phase of the material). Moreover, by comparing the two groups of curves, it can be found that: first, although the data in Figure 8 shows that the resistivity of group C is higher than that of group S at the same age in the case of unsaturated conditions, the resistivity of group S is higher than that of group C at the same moisture content. According to Equation (6), porous materials’ conductivity under unsaturated conditions is still positively correlated with porosity and pore connectivity, which is consistent with the actual results; second, both curves show that with the gradual decrease of material moisture content, the resistivity increases more and more. The reason is that with the decrease of water content, the weight of the high resistance part becomes increasingly higher, which leads to the increasing range of resistivity.

### 3.3. Pore Diameter Distribution

**Figure 12:**
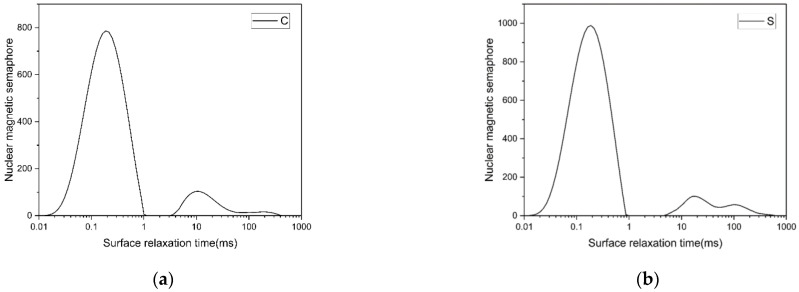
Surface relaxation time distribution curves of (**a**): group C and (**b**): group S.

Low-field NMR tests were performed on the two groups of samples after curing for 28 days (surface relaxation time distribution is shown in Figure 12). The results showed three peaks around 0.1 ms, 10 ms and 100 ms for both groups of samples. According to previous researchers’ experience, pore radius can be calculated by relaxation time T_2_ and surface relaxation ρ_2_. However, the surface relaxation of the material is difficult to calculate accurately, and it is usually obtained indirectly by referring to other methods such as MIP [27,28].

**Figure 13:**
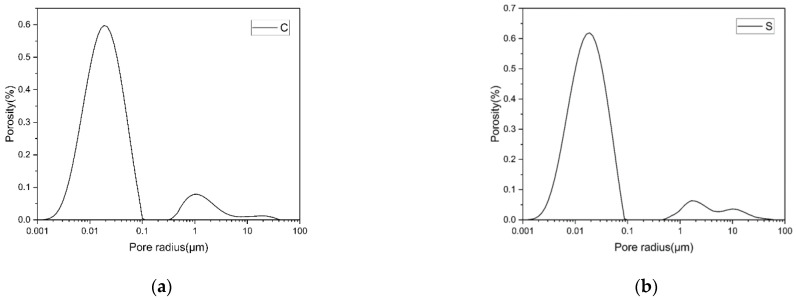
Pore size distribution curves of (**a**): group C and (**b**): group S.

**Figure 14:**
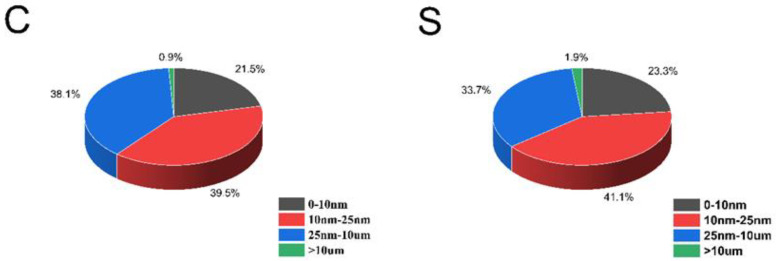
The porosity at different diameter ranges.

To compare the pore size distribution of the two groups of specimens, the empirical value of ρ2 is 50 μm/s in this paper, and the pore radius distribution can be calculated according to the fast exchange hypothesis [29,30,31], as shown in Figure 13. It can be seen that the pore size distribution curve of group S has three prominent peaks, centered at 18 nm, 1.6 μm and 10 μm, respectively. The pore size distribution curve of group C has two obvious peaks, centered at 20 nm and 1 μm, and there is a weak peak at 20 μm. Compared with group C, group S had smaller pores and larger macropores. Moreover, the maximum aperture of group S is smaller than that of group C. The porosities at different diameter ranges are shown in Figure 14 in order to better compare the pore size distribution of the two groups of samples. Compared with group C, there are more pores with pore sizes less than 25 nm in group S, indicating that silica fume plays a role in refining pores in the aluminate cement system. The number of coarse pores larger than 10 μm in group S is more than that in group C, which may be because the incorporation of silica fume leads to a higher cement paste consistency, making it more challenging to remove pores during molding, thus generating more coarse pores.

The total porosity of the samples was also obtained by a low-field NMR test. The experimental results showed that group C’s porosity was 6.74%, and that of group S was 7.89%. From the above experimental results, it can be seen that group C’s moisture content decreased faster than that of group S, and the resistivity of group S was higher than that of group C under the same moisture content. Combined with the pore size distribution of the two groups of samples, it can be concluded that compared with material’s porosity, the connectivity of pore channels is the most critical factor in determining the water content and resistivity of ACA.

## 4. Conclusions

The results indicated that the addition of silica fume improved the resistivity of ACA under the saturated state. This was mainly attributed to the decrease of the ACA’s pore connectivity due to the SF’s filling effect. However, the early compressive strength of ACA was slightly reduced by the addition of SF. Under the unsaturated state, the ACA’s resistivity without the SF gradually exceeded that with the SF as the extension of drying time. The results also indicated that the addition of SF content increased the ACA’s porosity; the tiny pores (the size less than 25 nm) especially increased by 3.4%. Meanwhile, the addition of SF increased the tortuosity of the ACA’s conductive channels. These factors can improve the resistivity of cement-based adhesives. Therefore, SF is recommended to be used in cement-based adhesives in insulators to lower costs and improve the resistivity.

## Figures and Tables

**Table 1:** Chemical composition of calcium aluminate cement and silica fume (mass%).

Composition	SiO_2_	Al_2_O_3_	Fe_2_O_3_	CaO	MgO	K_2_O	Na_2_O	TiO_2_	I.L
aluminate cement	4.45	52.03	1.71	37.53	0.76	0.25	0.07	2.54	0.20
SF	98.57	0.35	0.12	0.21	0.17	0.16	0.06		0.18

## Data Availability

Data is contained within the article.
